# Development of an Enzyme-Linked Immunosorbent Assay Based on a Monoclonal Antibody for the Rapid Detection of Citrinin in Wine

**DOI:** 10.3390/foods14010027

**Published:** 2024-12-25

**Authors:** Xingdong Yang, Yang Qu, Chenchen Wang, Lihua Wu, Xiaofei Hu

**Affiliations:** 1 Institute of Food and Drug Inspection, College of Life Science and Agronomy, Zhoukou Normal University, Zhoukou 466001, China; zkskyyxd@163.com (X.Y.); 13525010355@163.com (Y.Q.); 18403784292@163.com (C.W.); 18336897154@163.com (L.W.); 2 Key Laboratory of Animal Immunology, Ministry of Agriculture and Rural Affairs & Henan Provincial Key Laboratory of Animal Immunology, Henan Academy of Agricultural Sciences, Zhengzhou 450002, China

**Keywords:** citrinin, monoclonal antibody, *ic*-ELISA, wine

## Abstract

The ingestion of food contaminated with citrinin (CIT) poses a variety of health risks to humans and animals. The immunogens (CIT-COOH-BSA, CIT-H-BSA) and detection antigen (CIT-COOH-OVA, CIT-H-OVA) were synthesised using the active ester method (-COOH) and formaldehyde addition method (-H). A hybridoma cell line (3G5) that secretes anti-CIT monoclonal antibodies (mAbs) was screened via CIT-H-BSA immunisation of mice, cell fusion, and ELISA screening technology. The cell line was injected intraperitoneally to prepare ascites. The reaction conditions for the indirect competitive ELISA (*ic*-ELISA) were optimised, and an *ic*-ELISA method for detecting CIT was preliminarily established. The results revealed that the IC_50_ of CIT from optimised *ic*-ELISA was 37 pg/mL, the linear detection range was 5.9~230 pg/mL, and the cross-reaction (CR) rate with other analogues was less than 0.01%. The intra-assay and interassay sample recovery rates of CIT were 84.7~92.0% and 83.6~91.6%, and the coefficients of variation (CVs) were less than 10%. The *ic*-ELISA of CIT established in this study was not significantly different from the HPLC results and is rapid, highly sensitive and strongly specific, providing technical support for the detection of CIT.

## 1. Introduction

Citrinin (CIT) is a mycotoxin produced by *Monascus*, *Penicillium*, and *Aspergillus* fungi. CIT is widely found in *Monascus* products, mould grains, fruits, vegetables, herbs, spices, and related products [1]. CIT has multiple toxic effects on humans and animals, and is toxic to the liver, kidneys, heart, and gastrointestinal tract, among which hepatotoxicity and reproductive toxicity are the most obvious [2,3,4]. CIT also demonstrates genotoxicity and carcinogenicity [5]. Wine is an alcoholic beverage made from wine grapes and fermented over a long period of time. Wine grapes are easily contaminated by various moulds during growth, harvest, storage and fermentation process [6,7]. Among them, *Botrytis cinerea*, *Penicillium*, and *Aspergillus* are the moulds commonly isolated from grapes. Some of these moulds isolated and identified from wine grapes are consistent with citrinin toxin-producing fungi [8,9]. Inadvertent ingestion of such CIT-contaminated products can cause human illness or death and pose a great threat to human health. However, the toxicity mechanism of citrinin has not been fully clarified, which has prevented relevant agencies from correctly formulating accurate limits for citrinin in various types of food and feed [10].

The European Food Safety Authority (EFSA) noted that more research data are needed to ensure the accuracy of risk assessment [11]. The European Union, Republic of Korea, and Japan have set the maximum residue limits of citrinin in red yeast rice food supplements at 2000 μg/kg, 50 μg/kg, and 200 μg/kg [12]. At present, China has no limit values for citrinin in grains, oils, and related products, but has issued and implemented the national standard detection method for citrinin in different food matrices (GB 5009.222-2016) [13]. For example, the limits of detection (LODs) and limits of quantitation (LOQs) of CIT in rice, corn, and chili powder samples obtained via immunoaffinity column cleanup–high-performance liquid chromatography (HPLC) are 8 μg/kg and 25 μg/kg, respectively; the LODs and LOQs of red yeast and its products are 25 μg/kg and 80 μg/kg, respectively. The LOD and LOQ of CIT determined by C18-solid phase extraction cartridge cleanup–HPLC are 3 μg/kg and 10 μg/kg, respectively.

To date, HPLC [14], ultra-HPLC coupled with tandem mass spectrometry (UHPLC-MS/MS) [15], HPLC-fluorescence detection (HPLC-FLD) [16,17], gas chromatography (GC) [18], thin-layer chromatography (TLC) [19], and enzyme immunoassays [20] have been widely used to detect CIT. However, these types of methods have several limitations, namely, requiring a long time, expensive equipment, complex sample preparation, and the need for professional operators. To overcome these difficulties, immunoassays based on antigen–antibody recognition, such as direct competition enzyme–linked immunosorbent assay (dc–ELISA), indirect competitive ELISA (ic–ELISA) (Table 1), and lateral flow immunoassay (LFIA), are rapid, simple, and economical [21] and have been widely used in the detection of the small molecule CIT and other residues [13,22,23,24,25,26,27,28]. Compared with LFIA, ELISA can detect analytes qualitatively and quantitatively and is more accurate. However, there are no reports on the detection of citrinin residues in wine via this method.

In this study, CIT was coupled to different carrier proteins and the best paired antiserum, which were subsequently screened via antigen coating after immunising the mice. A hybridoma cell line (3G5) that secretes anti-CIT monoclonal antibodies (mAbs) was constructed via cell fusion technology. Using these antibodies, an *ic*-ELISA protocol was established to detect CIT in wine, with the aim of providing a new method for the detection of CIT in wine to control the legal residue of CIT in wine.

## 2. Materials and Methods

### 2.1. Chemicals and Materials

Citrinin standard, N-hydroxy succinimide (NHS) (purity 98%), 1-ethyl-3(3-dimethylaminopropyl) carbodiimide (EDC), Freund’s complete adjuvant (FCA), Freund’s incomplete adjuvant (FIA), hypoxanthine-aminopterin-thymidine (HAT), hypoxanthine-thymidine (HT), and anti-mouse horseradish peroxidase (HRP)-IgG were obtained from Sigma (St. Louis, MO, USA); patulin, aflatoxin B1, salbutamol, zearalenone, T-2 toxin, and deoxynivalenol (DON) were purchased from Macklin (Shanghai, China); bovine serum albumin (BSA), Ochratoxin A (OTA), and ovalbumin (OVA) were purchased from Aladdin (Shanghai, China); dimethylformamide (DMF), Tween-20, and other agents (analytical grade) were purchased from Sinopharm Chemical Reagent Co., Ltd. (Shanghai, China); and wine was purchased at a local supermarket.

BALB/c mice (female, 8 weeks old) were provided by the Laboratory Animal Center of Zhengzhou University.

A UV spectrophotometer (UV2450) from Shimadzu, Kyoto, Japan, and a Scientific Multiskan FC enzyme labelling instrument from Thermo (Waltham, MA, USA) were used. A 3K-18 high-speed refrigerated centrifuge (St. Louis, MO, USA) and a CP214 electronic analytical balance were obtained from OHAUS (Parsippany, NJ, USA).

### 2.2. Preparation of Complete Antigen from CIT

**Figure 1:**
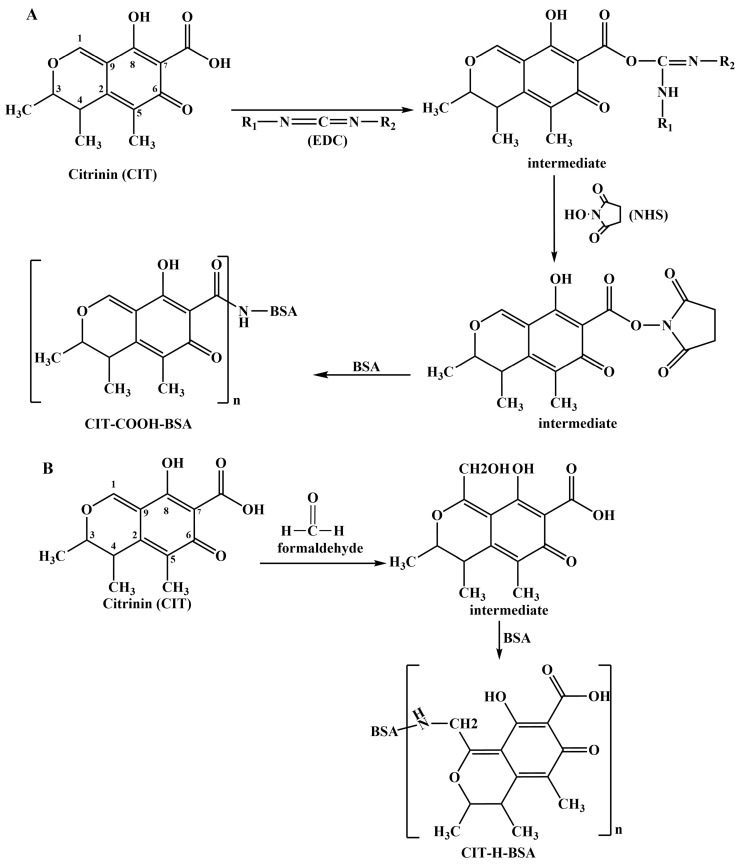
(**A**) Synthesis route of the complete antigen (citrinin-COOH-BSA); (**B**) synthesis of the complete antigen (citrinin-H-BSA).

Protein conjugation (active ester method) was based on the carboxyl group of CIT (Figure 1A) [26]. A total of 25.0 mg of citrinin was dissolved in 2.5 mL of DMF solution. Then, 11.5 mg of NHS and 15.5 mg of EDC were added, and the mixture was incubated at room temperature for 10 h (liquid A). Masses of 66.5 mg of BSA and 45.0 mg of OVA were dissolved in 2.5 mL of phosphate-buffered saline (PBS) to obtain the corresponding liquids B and C. In an ice bath, liquid A was slowly added to liquid B and C and the mixture was allowed to react at 4 °C for 12 h. The CIT-BSA and CIT-OVA conjugates were obtained. The conjugates were dialysed against PBS in dialysis bags at 4 °C for 72 h. The PBS was exchanged 6 times. The conjugates were stored at −20 °C for later use.

Protein conjugation (formaldehyde addition method) was based on the active hydrogen of CIT (Figure 1B) [22,28]. A total of 12.5 mg of citrinin was dissolved in 2.5 mL of methanol solution (solution A), and the conjugated protein (33.2 mg of BSA, 22.3 mg of OVA) was dissolved in 3 mL of 0.1 mmol/L sodium acetate solution (pH 4.2) (solution B). At room temperature, solution B was slowly added to solution A, followed by the addition of 4.0 mL of 37% formaldehyde solution and full mixing. The mixture was reacted at 37 °C for 48 h to obtain CIT-H-BSA and CIT-H-OVA conjugates. The conjugates were dialysed with 0.01 mol/L PBS at 4 °C for 72 h, during which the PBS was exchanged 6 times. The conjugates were then stored at −20 °C for later use.

### 2.3. Animal Immunisation and Cell Fusion

All experimental animals used in this study were approved by the Animal Ethics Committee of Zhoukou Normal University (ZKNU2023042). All methods were performed in accordance with the relevant guidelines and regulations. Six 8-week-old female BALB/c mice were divided into two groups. CIT-H-BSA and CIT-COOH-BSA were used as immunogens. The immunisation dose was 55.0 µg/mouse. The immunisation route involved multiple subcutaneous injections on the back. The immunisation interval was 22 days, and a total of 5 immunisations were performed. FCA with the same amount as the immunogen was used for the first immunisation, and FIA with the same amount as the immunogen was used for the 2nd to 5th immunisations. The mixture of the immunogen and Freund’s adjuvant was fully emulsified for 7 min. Two weeks after the 5th immunisation, the mice were tail-cut, and blood was collected for polyclonal antibody (pAb) serum. The ELISA method was used to identify the titre and sensitivity of pAb in the blood serum [21]. Mice with better immunological performance were screened. A total of 55.0 µg of immunogen was injected intraperitoneally 3 days before cell fusion for superstrong immunisation.

At the end of all the immunisation protocols, the mice were anaesthetised with isoflurane for three minutes followed by cervical dislocation. The spleens of the immunised mice were collected under sterile conditions, separated into single dispersed cells, fused with SP2/0 cells using PEG2000, resuspended in HAT medium, evenly plated in a 96-well cell culture plate, and cultured at 37 °C in a 5% CO_2_ environment. Half of the medium was replaced every 6 days, and after 8 days, an ELISA was carried out using the supernatant. The screened cell line was then subcloned three times via the limiting dilution method to obtain hybridoma cells that could stably secrete anti-CIT mAbs. The in vivo *ascites induction* method was selected to prepare anti-CIT mAbs. The affinity constant (*Ka*) [29] can be calculated according to the following formula:

*Ka* = (*n* − 1)/2(*n*[Ab’]*t* − [Ab]*t*)

*n* = [Ag]*t*/[Ag’]*t*

where [Ag]*t* and [Ag’]*t* represent two different concentrations of CIT-H-OVA, and [Ab]*t* and [Ab’]*t* represent the corresponding concentrations of the anti-CIT mAb.

### 2.4. ELISA Procedure

The *ic*-ELISA was performed according to the reference method [28]. To coat, CIT-OVA was diluted to 0.6 μg/mL in carbonate buffer solution (CBS), added to a 96-well ELISA plate, and incubated at 37 °C for 2 h. To block, ELISA plates were washed four times with 0.01 mol/L PBS with 0.1% Tween-20, then 215 μL of 5% porcine serum was added to the wells and blocked at 37 °C for 1 h. After blocking, 55 μL of CIT standard solution and 55 μL of monoclonal antibody solution were added to the wells and the reaction was allowed to proceed at 37 °C for 16 min. After washing four times, 50 μL of anti-mouse HRP-IgG containing 5% porcine serum (1:1000) was added to each well and incubated at 37 °C for 32 min. After 6 washes, 55 μL of 3,3′,5,5′,-Tetramethylbenzidine (TMB) substrate solution was added to each well and incubated at 37 °C for 8 min. To terminate, 55 μL of 2 mol/L H_2_SO_4_ was added to each well and the optical density (OD) of each well solution at 450 nm was read using an ELISA reader.

### 2.5. Optimisation of ELISA Experimental Conditions

The optimal concentration for CIT-OVA and the working concentration of the anti-CIT mAb were determined via checkerboard titration. CIT-OVA was diluted to 2.4, 1.2, 0.6, 0.3, and 0.1 μg/mL with CBS and coated on the ELISA plate vertically. The anti-CIT mAb was diluted 1750, 3500, 7000, 14,000, 28,000, and 56,000 times with PBS and added to the coated ELISA plate horizontally. PBS was used as a blank control. The coating concentration and anti-CIT mAb dilution factor corresponding to the wells with an OD_450_ nm value of approximately 1.0 were selected as the optimal coating concentration and the optimal dilution factor of the mAb by microplate reader detection.

Determination of the optimal reaction conditions for four factors was performed via orthogonal experiments. (1) The CIT-H-OVA coating conditions included incubation at 4 °C overnight, incubation at 37 °C for 1 h, incubation at 37 °C for 2 h, and incubation at 37 °C for 4 h. (2) The reaction times between the anti-CIT mAb and CIT-H-OVA at 37 °C were set to 8 min, 16 min, and 24 min. (3) The anti-mouse HRP-IgG was diluted 400, 1000, 1600, and 2200 times with blocking solution. (4) The colour development times were set to 4 min, 8 min, 12 min, and 16 min. The positive well/negative well (P/N) value was calculated according to the OD_450_ nm value via ELISA detection to determine the optimal experimental conditions. The P/N ratio is positively correlated with the experimental results.

### 2.6. Sample Treatment

A total of 4.0 mL of each wine sample was pipetted and weighed accurately (accurate to 0.01 g). Four millilitres of ammonium acetate solution (0.025 mol/L, pH 9.0) was added, the mixture was mixed 1:1, vortexed for 2 min, centrifuged at 7500 r/min for 6 min, filtered with a 0.22 μm filter membrane, and then tested.

### 2.7. Performance Evaluation of the ELISA Method

CIT standard solutions of different concentrations (160, 80, 40, 20, 10, 5, 2.5, and 0 pg/mL) were prepared as inhibitors, and anti-CIT mAbs with an OD_450_ nm value of approximately 1.0 were added to each well. Indirect competitive ELISA was used for detection, where the IC_50_ is the concentration that inhibits 50% of the binding of CIT to the anti-CIT mAb. The quantitative working range was set to the concentration that inhibits 20−80% of CIT binding to the antibody.

The CIT standard was added to the blank wine sample at concentrations of 365.0, 730.0, and 1095.0 pg/mL, and this process was repeated 6 times. Evaluation of the accuracy and precision of the *ic*-ELISA method was by recovery rate and intra-assay and interassay variation. The concentration of CIT in the sample was detected via *ic*-ELISA, and the spiked recovery rate and coefficient of variation were calculated.

Cross-reaction (CR) was used to evaluate *ic*-ELISA selectivity. Under the optimal working concentration conditions, seven CIT structural analogues, including patulin, aflatoxin B1, salbutamol, zearalenone, T-2 toxin, DON, and OTA were selected for cross-reaction experiments. Inhibition curves were drawn, and their corresponding IC_50_ values were calculated. The CR rate was calculated according to the following formula: CR/% = IC_50_ CIT/IC_50_ CIT structural analogues × 100%.

### 2.8. Comparative Analysis of ic-ELISA and HPLC Methods

Standard solutions of CIT at concentrations of 1100.0, 2200.0, and 3300.0 pg/mL were prepared. *ic*-ELISA and HPLC methods were used to determine the accuracy of *ic*-ELISA. The HPLC (Dima, Beijing, China) chromatographic conditions were as follows: a 20 µL sample was injected into a Diamonsil C18 (250 × 4.6 mm × 5 μm) column at 28 °C, with a mobile phase of acetonitrile and ultrapure water (67–33) at a flow rate of 1.0 mL/min; products were excited at 331 nm and emission was measured at 500 nm.

### 2.9. Data Processing

Excel 2019 was used for tables; GraphPad Prism 8 was used to generate the inhibition curve; Chem Draw Pro 22 was used for chemical structures.

## 3. Results

### 3.1. Identification of Artificial Antigen

**Figure 2:**
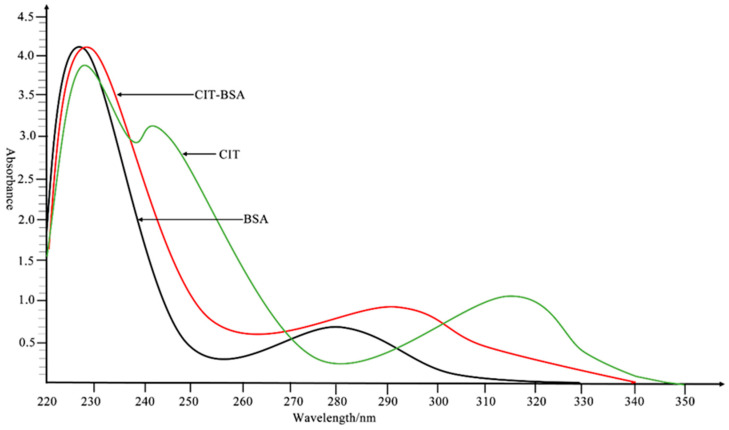
Ultraviolet spectrum of citrinin-H-BSA. The solvent was PBS (pH 7.4).

CIT (molecular weight of 250 daltons) is a small molecule that can be immunogenic only when linked to a carrier protein. CIT-COOH-BSA and CIT-H-BSA were identified via ultraviolet (UV) scanning to determine whether CIT and the carrier protein were successfully linked. In the range of 220–350 nm, the maximum UV absorption wavelengths of citrinin and BSA were measured by UV scanning to be 319 and 280 nm. Compared with that of BSA, the UV absorption peak of the coupled artificial antigen shifted (Figure 2), which proved that citrinin was linked to the carrier protein. BALB/c mice were immunised with CIT-COOH-BSA or CIT-H-BSA. After five immunisations, anti-CIT pAbs were obtained. The titres of the pAbs (CIT-H-BSA) were 2.56 × 10^4^, 1.28 × 10^4^, and 2.56 × 10^4^ according to indirect ELISA. The titres of the pAbs (CIT-COOH-BSA) were 1.28 × 10^4^, 1.28 × 10^4^, and 2.56 × 10^4^ (Table S1). The sensitivity of the pAbs was determined via *ic*-ELISA. The IC_50_ values of pAbs (CIT-H-BSA) were 6.22, 27.71, and 13.49 ng/mL, respectively (Table S2). Although CIT-COOH-BSA-immunised mice produced pAbs with good titres, no specific pAbs against citrinin were detected. Therefore, mouse no. 1 immunised with CIT-H-BSA could be further used to prepare mAbs (Table 2).

### 3.2. Anti-CIT mAb Assay Results

After the cell fusion experiments, a total of four hybridoma cell lines that could stably secrete anti-CIT mAbs were obtained: 1E8, 2D7, 3G5, and 4A2. The IC_50_ values of the mAbs secreted by the cell lines 1E8, 2D7, 3G5, and 4A2 were determined via *ic*-ELISA (using CIT-H-BSA as the coating antigen) (Tables S3 and S4). Among them, the antibody secreted by the 3G5 cell line had the highest potency (1:5.12 × 10^5^) and sensitivity, and the affinity constant (Kaff) was 1.52 × 10^10^ L/mol (Table S5).

### 3.3. Selection of the Best Experimental Conditions for ic-ELISA

The checkerboard titration method assayed different coating-antigen (CIT-H-OVA) coating concentrations and different anti-CIT mAb working concentrations for detection by indirect ELISA. The CIT-OVA mass concentration of 0.6 μg/mL and anti-CIT mAb dilution of 1:28,000 yielded an indirect ELISA value of 1.019. As this was the value closest to 1.0 by a large margin, these concentrations were selected as the optimal experimental conditions (Table S6).

**Figure 3:**
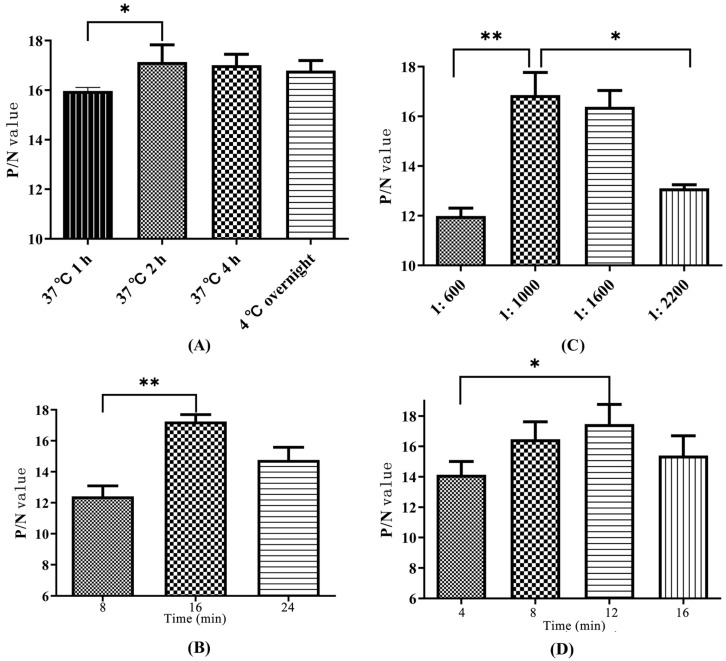
(**A**) Coating conditions of citrinin-H-OVA. (**B**) Reaction time of citrinin and anti-citrinin mAb. (**C**) Dilution ratio of the anti-mouse HRP-IgG. (**D**) Colour development time. “*”: the difference is significant (*p* < 0.05), “**”: the difference is very significant (*p* < 0.01).

The results of the four-factor orthogonal test are shown in Figure 3. The experimental conditions corresponding to the P/N values for each factor are selected as the best. (1) The P/N value corresponding to 2 h at 37 °C was slightly greater than that corresponding to overnight at 4 °C and 4 h at 37 °C (*p* > 0.05), but significantly greater than P/N values corresponding to 1 h at 37 °C (*p* < 0.05). After comprehensive consideration, 2 h at 37 °C was selected as the coating condition of the CIT-H-OVA (Figure 3A). (2) The P/N value corresponding to the reaction time between CIT-H-OVA and the anti-CIT mAb for 16 min was greater than that for 8 min, and the difference was significant (*p* < 0.01), and there was no significant difference in the P/N values corresponding to 24 min (*p* > 0.05). Sixteen minutes was selected as the optimal antigen–antibody reaction time (Figure 3B). (3) The P/N value corresponding to the dilution multiple of the anti-mouse HRP-IgG of 1000 was the most ideal, and a 1000-fold dilution was selected as the optimal dilution concentration of the anti-mouse HRP-IgG (Figure 3C). (4) The P/N value corresponding to the colour development time of 12 min was greater than that of 4 min, 8 min, and 16 min, but there was no significant difference between them (*p* > 0.01). A time of 12 min was selected as the optimal colour development time (Figure 3D).

### 3.4. Performance Evaluation of the Established ic-ELISA

**Figure 4:**
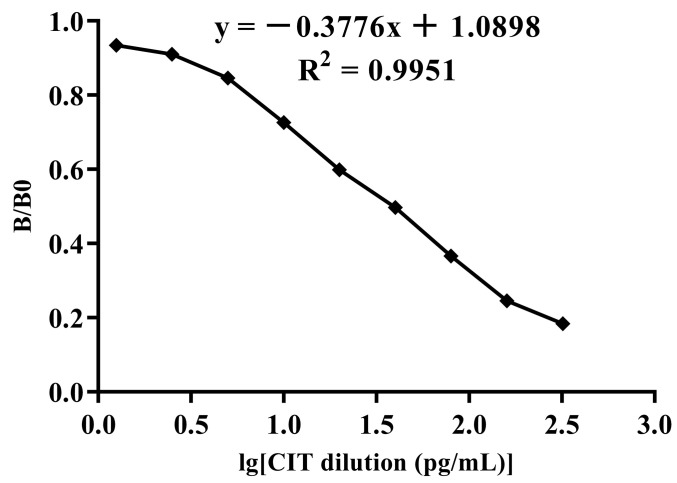
Standard curve of indirect competitive ELISA for anti-citrinin mAbs.

In accordance with the results of the checkerboard titration method, the ELISA plate was coated with CIT-OVA at a concentration of 0.6 μg/mL, and the anti-CIT mAb was diluted 1:28,000 and loaded. The optimised *ic*-ELISA standard curve is shown in Figure 4. The results showed that the *ic*-ELISA inhibition curve had good linearity; the regression equation was y = −0.3776x + 1.0898 (R^2^ = 0.9951), the IC_50_ value was 37 pg/mL, and the linear detection range of the standard curve was calculated to be 5.9~230 pg/mL.

The *ic*-ELISA method was used to detect the CIT content in wine samples supplemented with CIT standards, and the CIT recovery rate in the actual samples was calculated. The results revealed that the recovery rate and precision of CIT reached a high level. The data results are shown in Table 3 and Table S7. After detection and analysis, the intra-assay and interassay recoveries of CIT were 84.7~92.0% and 83.6~91.6%, respectively; the CV of the interassay experiment was greater than that of the intra-assay experiment and was less than 10%. The results show that the established CIT *ic*-ELISA has a high recovery rate for CIT analysis and can be used to accurately analyse the CIT content in wine samples.

The CRs of the anti-CIT mAb with CIT and the other seven structural analogues of CIT (patulin, aflatoxin B1, salbutamol, zearalenone, T-2 toxin, DON, and OTA) were analysed. The results in Table 4 show that the reaction rate of the anti-CIT mAb with CIT was 100%, while it had no CR with the seven structural analogues of CIT, indicating that the prepared *ic*-ELISA has high selectivity for CIT.

### 3.5. Comparison Between ic-ELISA and HPLC

The *ic*-ELISA detection method established in this study and the HPLC method were used to detect the samples at the same time. The detection results are shown in Table 5 and Table S8. The results revealed that the difference in the detection results of the two methods was not significant, indicating that the ELISA method established in this study has high reliability.

## 4. Conclusions

In this study, two conjugates of CIT and a carrier protein (BSA) (CIT-COOH-BSA and CIT-H-BSA) were prepared via the active ester method and formaldehyde addition method. According to the experimental data analysis, two conjugation reactions of CIT with proteins were successful, but the results of the *ic*-ELISA experiments revealed that the colour reaction of the immune serum of the conjugate (CIT-BSA) connected by a carboxyl group could not be blocked by CIT molecules. It is inferred that the carboxyl group of citrinin is an important group in its antigenic determinant. If the carboxyl group of CIT is coupled to the protein, the antigenicity of citrinin may change and it may not effectively induce the body to produce antibodies against citrinin molecules. The conjugate (CIT-H-BSA) did not use the carboxyl group of CITs as a connecting group but prepared the CIT conjugate through the active hydrogen at the C1 position on the CIT molecule. Therefore, CIT-H-BSA stimulates the body to produce specific antibodies against CIT.

Mice were immunised with CIT-H-BSA and anti-CIT mAbs were obtained via combination with cell fusion technology. The optimal CIT-OVA coating concentration was determined to be 0.6 μg/mL and the working concentration of the anti-CIT mAb was 1:28,000 dilution, as determined via the checkerboard titration method. Moreover, the optimal conditions for the *ic*-ELISA reaction were as follows: coating conditions (2 h at 37 °C), CIT and anti-CIT mAb reaction time (16 min), anti-mouse HRP-IgG dilution ratio (1:1000), and colour development time (8 min). The *ic*-ELISA method for detecting CIT was established, with an IC_50_ of 37 pg/mL and a linear range of 5.9~230 pg/mL. This method has good selectivity, and the CR of the other seven structural analogues is less than 0.01%. The intra-assay and interassay recoveries of CIT in spiked wine samples were 84.7~92.0% and 83.6~91.6%, respectively, and the relative standard deviations of intra-assay and interassay tests were less than 10%, indicating that the *ic*-ELISA detection performance is good and can be used for the detection of CIT in wine. This study has great significance for the rapid and accurate determination of the CIT content in wine by establishing a CIT *ic*-ELISA, which provides a scientific method for the supervision and inspection of illegal or excessive addition of CIT in food and has high practical application value.

## Figures and Tables

**Table 1:** Published immunoassays for the determination of citrinin.

Immunoassay	Antibody Type	IC_50_ (ng/mL)	Linear Range (ng/mL)	Limit of Detection (ng/mL)	Samples	References
*ic*-ELISA	pAb	0.41	-	0.04	wheat, corn flour	[22]
*ic*-ELISA	pAb	-	20–640	10	buffer	[23]
*dc*-ELISA	pAb	4.1	0.5–36.6	0.2	red yeast rice	[24]
*dc*-ELISA	pAb	5.0	-	0.2	red yeast rice	[25]
*ic*-ELISA	mAb	200.0	20–1000	10	wheat	[26]
*ic*-ELISA	mAb	200	20–500	0.4	grain, corn	[27]
*ic*-ELISA	mAb	0.28	0.01–5.96	0.01	red yeast rice, cheese, wheat bread, apple	[28]
*ic*-ELISA	mAb	0.037	0.059~0.23	0.059	wine	This work

**Table 2:** Titre and sensitivity of anti-citrinin polyclonal antibodies.

Methods for Synthesising Antigens	Immunised Mouse Serial Number	Titre of pAbs	IC_50_ of pAbs (ng/mL)
Active ester method (CIT-COOH-BSA)	1	2.56 × 10^4^	Not detected
	2	1.28 × 10^4^	Not detected
	3	2.56 × 10^4^	Not detected
Formaldehyde addition method (CIT-H-BSA)	1	1.28 × 10^4^	27.71
	2	1.28 × 10^4^	13.49
	3	2.56 × 10^4^	6.22

**Table 3:** The recovery rate of CIT in wine samples.

Spiked CIT (pg/mL)	Intra-Assay			Interassay		
	Mean ± SD (pg/mL)	Recovery (%)	CV (%)	Mean ± SD (pg/mL)	Recovery (%)	CV (%)
365	310 ± 22	84.9 ± 6.0	7.1	310 ± 25	84.9 ± 6.8	8.1
730	660 ± 38	90.4 ± 5.2	5.9	660 ± 43	90.4 ± 5.9	6.4
1095	1010 ± 44	92.2 ± 4.0	4.3	1000 ± 50	91.6 ± 4.6	4.8

**Table 4:** Cross-reactivity of the anti-citrinin mAb to its structural analogues.

Compound	Chemical Structure	IC_50_ (pg/mL)	Cross-Reaction (%)
CIT	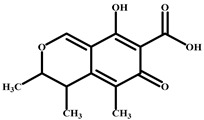	37	100
Patulin	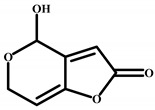	>4.0 × 10^3^	<0.01
Aflatoxin B1	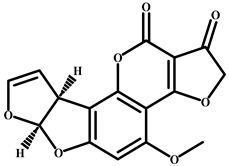	>4.0 × 10^3^	<0.01
Salbutamol	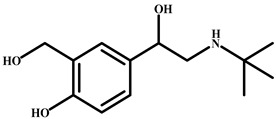	>4.0 × 10^3^	<0.01
Zearalenone	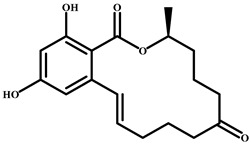	>4.0 × 10^3^	<0.01
T-2 toxin	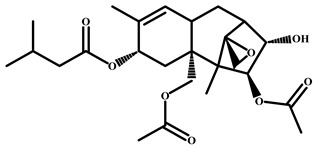	>4.0 × 10^3^	<0.01
Deoxynivalenol	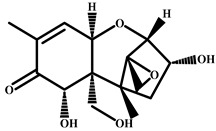	>4.0 × 10^3^	<0.01
Ochratoxin A	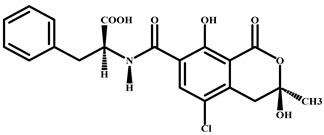	>4.0 × 10^3^	<0.01

**Table 5:** Comparison of the results of HPLC and indirect competitive ELISA established in this study.

Level of CIT (pg/mL) in Wine Samples	*ic*-ELISA (pg/mL)	CV	HPLC (pg/mL)	CV
Low—1100	1010 ± 42 *^a^*	4.2	1040 ± 38 *^a^*	3.7
Medium—2200	2050 ± 81 *^a^*	4.0	2090 ± 51 *^a^*	2.4
High—3300	3110 ± 80 *^a^*	2.6	3210 ± 74 *^a^*	2.3

*a* The superscript represents no statistical significance between the results given by the *ic*-ELISA and HPLC (*p* > 0.05).

## Data Availability

The data presented in this study are available on request from the corresponding author.

## Supplementary Materials

The following supporting information can be downloaded at: https://www.mdpi.com/2304-8158/14/1/27/s1, Table S1: Titres of polyclonal antiserum against citrinin; Table S2: Determination of sensitivity of polyclonal antiserum against citrinin; Table S3: The indirect ELISA titre of monoclonal antibodies against citrinin; Table S4: Inhibitive titres and inhibition curves of monoclonal antibodies against citrinin; Table S5: Determination of affinity constants for monoclonal antibody against citrinin; Table S6: Selection of coating concentration of the citrinin-H-OVA and dilution ratio of the monoclonal antibody; Table S7: Recovery of the indirect competitive ELISA for citrinin spiked in wine samples; Table S8: Comparison of the indirect competitive ELISA with HPLC of citrinin residues in wine samples.
