# Supplementary material for: Development of an Enzyme-Linked Immunosorbent Assay Based on a Monoclonal Antibody for the Rapid Detection of Citrinin in Wine
Source: Foods. 2024 Dec 25;14(1):27. doi: 10.3390/foods14010027 (PMC12068006; doi:10.3390/foods14010027)
Supplement: Supplementary file 1 [file foods-14-00027-s001.zip › Table S6 Selection of coating concentration of the citrinin-H-OVA and dilution ratio of the monoclonal antibody.pdf]

Table S6. Selection of coating concentration of the citrinin-H-OVA and dilution ratio of the mono-clonal antibody.

Coating concentration of CIT-H-OVA	Dilution ratio of the anti-CIT mAb							
	1750	3500	7000	14000	28000	56000	BC	NC
2.4 μg/mL	2.965	2.851	2.686	2.413	2.149	1.725	0.079	0.072
1.2μg/mL	2.615	2.296	2.212	1.871	1.625	1.232	0.074	0.069
0.6μg/mL	2.176	1.917	1.782	1.325	1.019	0.844	0.080	0.075
0.3μg/mL	1.681	1.348	1.230	1.112	0.874	0.419	0.074	0.066
0.1μg/mL	1.229	0.814	0.691	0.428	0.342	0.164	0.072	0.063
